# Supplementary material for: Investigating Connectivity Gradients in Schizophrenia: Integrating Functional, Structural, and Genetic Perspectives
Source: Brain Sci. 2025 Feb 11;15(2):179. doi: 10.3390/brainsci15020179 (PMC11853694; doi:10.3390/brainsci15020179)
Supplement: Supplementary file 1 [file brainsci-15-00179-s001.zip › Supplementary Text S1.pdf]

Supplementary Text S1.

Gradients components

The primary-to-transmodal functional gradient explained 30.5 ± 5.2% of the total connectivity variance (schizophrenia: 30.3 ± 5.3%; controls: 30.7 ± 5.2%). For the primary-to-transmodal morphological similarity gradient (MSN), 25.2 ± 2.3% of the total variance was explained (schizophrenia: 25.6 ± 3.0%; controls: 24.6 ± 1.1%). The first two gradients were selected for analysis due to their ability to capture a significant number of variances while minimizing the number of components involved.

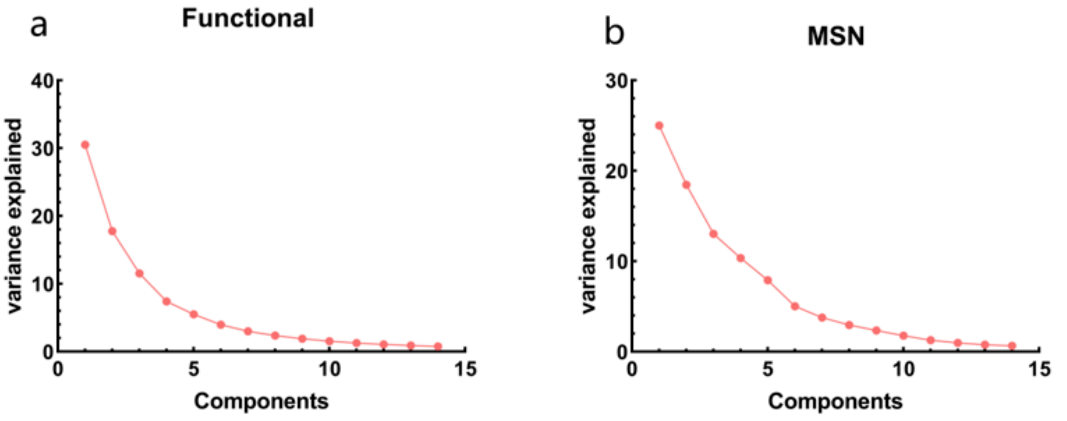

Figure S1. Fragmentation map. (A) Functional gradient of the fragmentation map. (B) Morphological similarity gradients of the fragmentation map.

Comparison of morphological similarity gradients

Significant differences were found between schizophrenic patients and controls on the morphological similarity gradient. In terms of the primary gradient of the functional gradient, the sensorimotor network (t=2.697, p<0.05) in schizophrenic patients showed lower gradient scores compared to controls. In terms of the secondary gradient of the morphological similarity gradient, the visual network (t=-4.554, p<0.05) in schizophrenics showed higher gradient scores compared to controls.

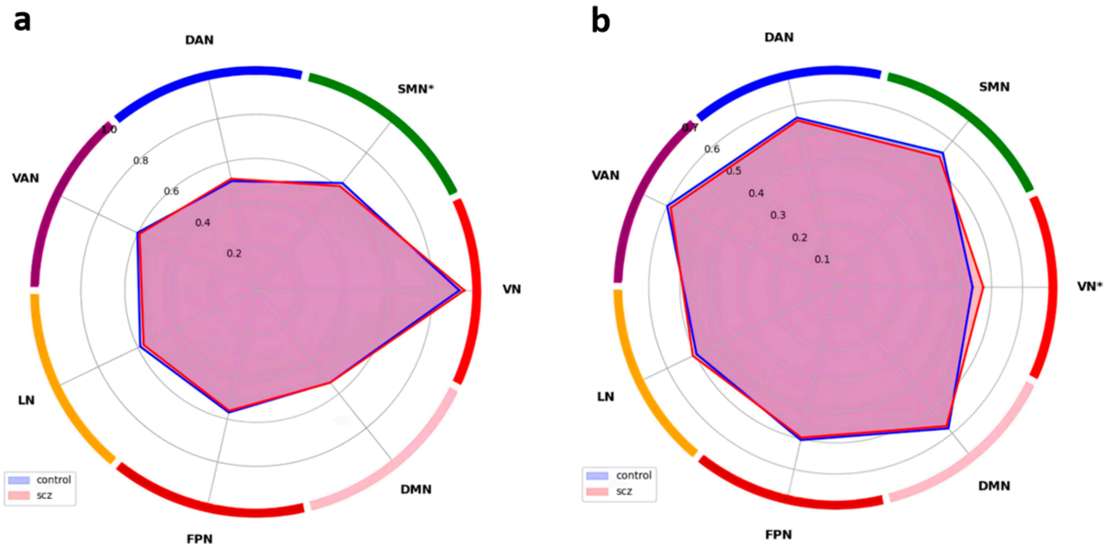

Figure S2. Radar-plots showing the Yeo-network profile of each group-level mean morphological similarity gradient. (A) Relative to controls, individuals with schizophrenia exhibited reduced scores on the morphological similarity principal gradient within the sensorimotor network. (B) Comparing with controls, individuals with schizophrenic demonstrated higher scores on the morphological similarity secondary gradient within the visual network.

PLS analysis of genetic data and functional gradients

The explained variation of the partial least squares principal components is shown in a of Figure S3. Since PLS1 and PLS2 typically capture the major part of the covariance between X and Y, we chose the first two principal components of partial least squares for our analysis. The first two components of the PLS regression explained 51.7% of the variance in SCZ-related changes in the primary gradient. A permutation test was performed on the first two principal components, and the results are shown in c, d of Figure S3. We computed confidence intervals for PLS and the results are shown in Figure S3, b. We calculated the VIP value for each gene and visualized the results for VIP values greater than 1, as in e of Fig. S3.

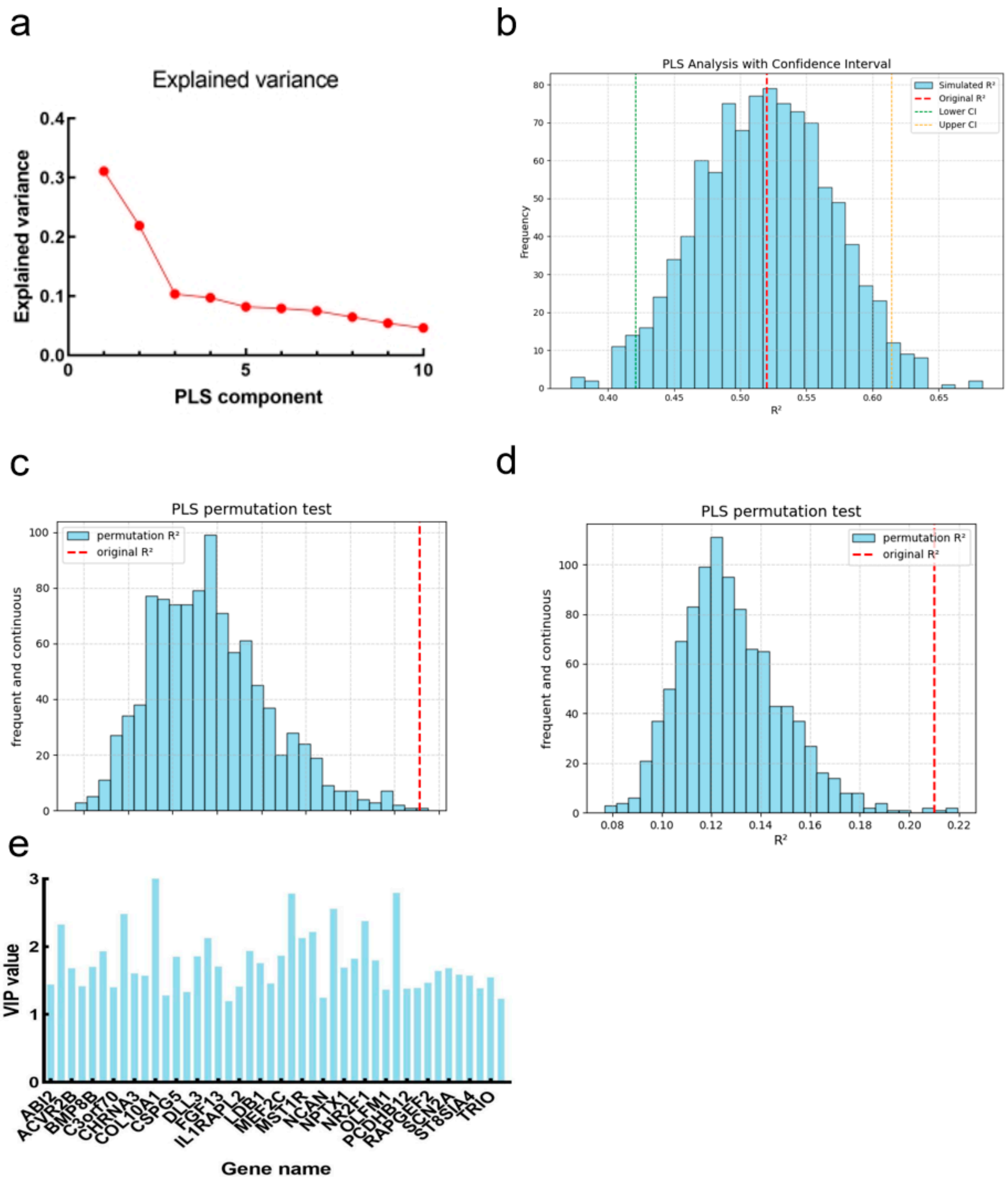

Figure S3. (A) The explained variation of the partial least squares principal components. (B)

Confidence intervals for PLS. (C) Permutation test for the first principal component of partial least squares. (D) Permutation test for the secondary principal component of partial least squares. (E) Genes with VIP values greater than 1.
