# Supplementary material for: Investigating Connectivity Gradients in Schizophrenia: Integrating Functional, Structural, and Genetic Perspectives
Source: Brain Sci. 2025 Feb 11;15(2):179. doi: 10.3390/brainsci15020179 (PMC11853694; doi:10.3390/brainsci15020179)
Supplement: Supplementary file 1 [file brainsci-15-00179-s001.zip › Supplementary Text S2.pdf]

Supplementary Text S2. Power analysis of gradient results and Enrichment of genes associated with schizotypy-associated hierarchy pattern

Table S1. Power analysis of gradient results

gradient	Effect size of each network	Statistical effectiveness of networks
The principal gradient of the functional gradient	Limbic network: -1.635<br>Frontoparietal control network: -0.925<br>Default mode network: -1.465<br>Visual network: 1.266	Limbic network: 1.000<br>Frontoparietal control network: 1.000<br>Default mode network: 1.000<br>Vision network: 1.000
The secondary gradient of the functional gradient	Sensorimotor network: 1.66<br>Limbic network: 0.695<br>Frontoparietal control network: 1.294<br>Default mode network: 1.856	Sensorimotor network: 1.000<br>Edge network: 0.986<br>Frontoparietal control network: 1.000<br>Default mode network: 1.000
The principal gradient of the morphological similarity gradient	Sensorimotor network: 1.629	Sensorimotor network: 1.000
The secondary gradient of the morphological similarity gradient	Visual network: 0.926	Visual network: 1.000

Table S2. Enrichment of genes associated with schizotypy-associated hierarchy pattern

GO term	Description	P	FDR q	Enrichment	Genes
Biological Process					
GO:0050808	synapse organization	2.69E-8	3.32E-4	3.67	TNR，SHANK3，ERC2，CACNB3，PPFIA4，EPHA4，PPFIA2，KALRN，PDZRN3，FARP1，SHANK1，SHANK2，GPM6A，LRRTM1，GPHN，NLGN1，KIRREL3，NLGN4X，PLXND1，DLGAP3，BSN，GRM5，PCDHGC5，FGF13，NRXN2，SYBU，CDH2
GO:0050804	modulation of chemical synaptic transmission	1.12E-7	6.9E-4	2.50	TNR，TSHZ3，SHISA9，SHANK3，DCC，ERC2，DLGAP，CACNB3，PTPRS，DLGAP4，EPHA4，PPFIA2，APBA1，SLC4A10，CALB1，PLK2，NPTX1，CSPG5，STX1A，NTNG1，MEF2C，ABR，AKAP5，SHANK1，SHANK2，JPH3，CAMK2A，NLGN1，GRIK2，NLGN4，HTR2A，GRIN2B，RAB3B，DLGAP3，GRM5，GRM7，CREB1，GRM8，CHRNA3，PRRT，NPTXR，RAPGEF2，CDH2
GO:0099177	regulation of trans-synaptic signaling	1.29E-7	5.31E-4	2.49	TNR，TSHZ3，SHISA9，SHANK3，DCC，ERC2，DLGAP1，CACNB3，PTPRS，DLGAP4，EPHA4，PPFIA2，APBA1，SLC4A10，CALB1，PLK2，NPTX1，CSPG5，STX1A，NTNG1，MEF2C，ABR，AKAP5，SHANK1，SHANK2，JPH3，CAMK2A，NLGN1，GRIK2，NLGN4X，HTR2A，GRIN2B，RAB3B，DLGAP3，GRM5，GRM7，CREB1，GRM8，CHRNA3，PRRT2，NPTXR，RAPGEF2，CDH2
GO:0050773	regulation of dendrite development	4.64E-7	1.15E-3	2.88	TIAM1，BHLHB9，ZDHHC15，CSMD3，ITPKA，SHANK3，DCC，PTPRS，EPHA4，NEURL，DLG5，PPFIA2，KIAA0319，ABI2，SARM1，PLK2，FSTL4，KALRN，MEF2C，SHANK1，SHANK2，NLGN1，KNDC1，CAMK1，HECW2，CDK5R1，CHRNA3，RAPGEF2，BCL11A，VLDLR
GO:0007399	nervous system developmen	7.17E-7	1.48E-3	2.20	BDNF，SCN3B，BZW2，OLFM1，SRRM4，CRMP1，NEURL，EVL，APBA1，MST1R，ABI2，DCX，TYRO3，

	t				FAM5C，SARM1，RAPGEFL1，ST8SIA4，CSPG5，AVIL，PCDHB12，KALRN，NR2F1，MYLIP，MERTK，MEF2C，DLG4，NUMBL，FGF17，CNTN3，GRIK1，NLGN1，C3orf70，ARID1A，NELL1，NR2C2，MET，NRSN2，CPNE6，CAMK1，SCN2A，MBD5，NRG4，DPYSL4，CHRNA3，FGF13，TCF4，LDB1，VLDLR
GO:0048731	system developmen t	1.47E-4	4.44E-2	1.61	PLEKHA5，BDNF，SCN3B，FRZB，CRMP1，TEK，NEURL，EVL，APBA1,IL1RAPL2,FAM5C,TYRO3,PPP1R9B,SARM1,NPTX1,ST8SIA4,CSPG5,PCDHB12,AVIL,KALRN,NR2F1,MYLIP,MEF2C,DLL3,DLG4,FGF17,BMP8B,CNTN3,C3orf70,NELL1,NR2C2,MET,CPNE6,MARCKS,CAMK1,SCN2A,COL10A1,NRG4,DPYSL4,CHRNA3,FGF13,TCF4,NCAN,TRIO,LDB1,RAPGEF2,PAPSS2,BZW2,OLFM1,SRRM4,ACVR2A,MST1R,ACVR2B,ABI2,DCX，RAPGEFL1，TLL1，SOX4，MERTK，NUMBL，WDR5，GRIK1，NLGN1，ARID1A，NRSN2，MBD5，MMP16，NDN，HPCAL4，CACNA1C，VLDLR
Component					
GO:0044456	synapse part	3.41E-10	5.62E-7	2.17	352DENND1A，SHISA9，ZNRF2，SHANK3，PTPRO，ERC2，PPFIA4，PTPRS，EPHA4，PPFIA2，APBA1，SLC4A10，CALB1，LRRC7，CSPG5，CAMK2N1，MEF2C，ABR，AKAP5，SHANK1，SHANK2，GPM6A，KCNA4，LRFN4，NRP2，GPHN，CAMK4，CAMK2A，MX2，ZNRF1，KCND3，RAB3B，ANKS1B，CAMK1，SCN2A，CDK5R1，CHRNA3，TENM2，NRXN2，BCL11A，CDH2，CDH8，DCC，DLGAP1，DLGAP4，DLG5，STRN4，MINK1，ABI2，CASK，STX1A，PRR12，NTNG1，GLRA2，FARP1，DAGLB，NR1D1，PDE4B，LRRTM1，NLGN1，KIRREL3，GRIK2，NLGN4X，GRIN2B，HTR2A，DLGAP3，CPEB4，CAP2，BSN，GRM5，GRM7，PRRT2，HOMER2，PDYN，

					SYT5，CACNA1C，CACNA1D.17.62
GO:0097458	neuron part	6.67E-9	5.5E-6	1.76	TRAK1，GPRIN1，KCNB2，DENND1A，ZNRF2，SHANK3，APBA1，SLC4A10，CAMK2N1，ABR，RGS8，GPM6A，SHANK2，KCNA4，GPHN，MYRIP，BRINP2，KCND3，ANKS1B，SCN2A，SCN3A，TENM2，FGF13，NRXN2，RAPGEF2，CDH2，CDH8，DCC，MINK1，DCX，PRR12，PDE4B，LRRTM1，DOCK4，GRIK2，NLGN4X，GRIN2B，DLGAP3，CPEB4，CCSAP，AGBL4，GRM5 ，GRM7，CREB1，PRRT2，HOMER2，PDYN，SYBU，CACNA1C，CACNA1D，AUTS2，NPHP1，ASTN1，SHISA9，PTPRO，ERC2，PPFIA4，PTPRS，EPHA3，EPHA4，PPFIA2，EPHA5，NPPA，CALB1，FAM5C，LRRC7，SARM1，SLC25A27，NPTX1，AKAP5，SHANK1，LRFN4，NRP2，CAMK2A，CAMK2D，MX2，EXOC6，ZNRF1，RAB3B，SYT13，CAMK1，CDK5R1，CHRNA3，STMN1，BCL11A，ACAP3，TSHZ3，DLGAP1，DLG5，ABI2，MYO7A，PLK2，CASK，STX1A，NTNG1，GLRA2，FARP1，NR1D1，FCHSD2，NLGN1，KIRREL3，ARID1A，HTR2A，NRSN2，HTR2C，CAP2，BSN，FAM161A，SYT5，LMTK3，PRPH2，CD200
GO:0043005	neuron projection	7.48E-9	4.11E-6	2.05	TRAK1，KCNB2，DENND1A ，SHANK3，PTPRO，PTPRS，EPHA3，EPHA4，EPHA5，PPFIA2，APBA1，SLC4A10，CALB1，FAM5C，SARM1，CAMK2N1，ABR，RGS8，SHANK1，SHANK2，GPM6A，KCNA4，NRP2，GPHN，CAMK2A，MX2，CAMK2D，BRINP2，KCND3，SYT13，ANKS1B，SCN2A，SCN3A，CDK5R1，CHRNA3，STMN1，TENM2，FGF13，RAPGEF2，CDH2，DCC，MINK1，ABI2，DCX，MYO7A，PLK2，STX1A，PRR12，GLRA2，FARP1，NR1D1，PDE4B，LRRTM1，DOCK4，KIRREL3，GRIN2B ，HTR2A，NRSN2，HTR2C，

					CPEB4，BSN，CCSAP，GRM5，GRM7，CREB1，PRRT2，HOMER2，PDYN，SYT5，LMTK3，CACNA1C，CD200，
GO:0043197	dendritic spine	4.47E-8	1.84E-5	3.78	PTPRO，SHANK3，EPHA4，STRN4，PPFIA2，APBA1，CALB1，ABI2，FARP1，ABR，AKAP5，NR1D1，SHANK1，PDE4B，GPM6A，SHANK2，KCNA4，CAMK2A，NLGN1，KCND3，ANKS1B，CPEB4，CDK5R1，PRRT2，TENM2
GO:0044309	neuron spine	4.47E-8	1.47E-5	3.78	PTPRO，SHANK3，EPHA4，STRN4，PPFIA2，APBA1，CALB1，ABI2，FARP1，ABR，AKAP5，NR1D1，SHANK1，PDE4B，GPM6A，SHANK2，KCNA4，CAMK2A，NLGN1，KCND3，ANKS1B，CPEB4，CDK5R1，PRRT2，TENM2
GO:0098794	postsynapse	4.94E-8	1.36E-5	2.72	TIAM1，ZDHHC15，ITPKA，GABRE，SHANK3，PTPRO，EPHA4，NEURL，STRN4，PPFIA2，APBA1，SLC4A10，CALB1，ABI2，FARP1，MEF2C，ABR，DLG4，AKAP5，NR1D1，PDE4B，SHANK1，GPM6A，SHANK2，KCNA4，CAMK4，CAMK2A，NLGN1，KCND3，ARHGAP32，ANKS1B，CPEB4，CDK5R1，PRRT2，WASF1，TENM2，GABRA2，CPT1C
GO:0099572	postsynaptic specialization	7.42E-8	1.75E-5	3.05	SHISA9，SHANK3，DLGAP1，DLGAP4，DLG5，MINK1，LRRC7，PRR12，STX1A，CAMK2N1，AKAP5，PDE4B，SHANK1，SHANK2，GPHN，CAMK2A，NLGN1，GRIK2，MX2，GRIN2B，KCND3，ANKS1B，DLGAP3，CAMK1，CPEB4，BSN，CAP2，CDK5R1，CHRNA3，HOMER2，BCL11A，CDH2，CACNA1C
